# Imaging a microfocus X-ray focal spot with a thin coded aperture

**DOI:** 10.1038/s41598-022-23338-y

**Published:** 2022-11-03

**Authors:** Graham R. Davis, Thomas Beckenbach, Pascal Meyer

**Affiliations:** 1 Institute of Dentistry, Queen Mary University of London, London, E1 4NS UK; 2 Microworks GmbH, Schnetzlerstr. 9, 76137 Karlsruhe, Germany; 3 Institute of Microstructure Technology, Karlsruhe Institute of Technology, Hermann-Von-Helmholtz-Platz 1, 76344 Eggenstein-Leopoldshafen, Germany

**Keywords:** Optics and photonics, Physics

## Abstract

Imaging of the focal spot size in X-ray generators can be achieved by means of a pinhole in a highly attenuating material, such as gold. For microfocus generators with spot sizes of only around 10 microns or less, the material must be very thin to avoid an impractical aspect ratio. With a 90 kV source, only 11% attenuation is attained with 5 µm gold. For a pinhole that is smaller than the focal spot, the signal-to-noise ratio (SNR) may be less than 1. To image the focal spot of a medical X-ray generator, a coded aperture has been used previously to reduce the exposure time required, however the same technique does not appear to have been used to increase the SNR when the attenuation is very low. Such a method is used here, using a no-two-holes-touching variation of a modified uniformly redundant array (MURA). In a prototype sample, with only 5 µm gold having 2.75 µm holes, the focal spot of a microfocus X-ray generator used in a micro-CT system could be clearly visualised and quantified. Directionality of the aberrations made focussing of the X-ray spot more intuitive and reduced the time required to obtain an optimal, quantifiable focus.

## Introduction

X-ray microtomography (micro-CT or XMT) is becoming increasingly popular in a variety of disciplines including materials science^1^, biology^2^, cultural heritage^3^, palaeontology^4^, and dentistry^5^, to name but a few. This diversity of applications has brought a huge increase in the use of microfocus X-ray technology and the ability to focus the electron beam in such equipment is essential for obtaining high quality images; the better the electron focus, the smaller the X-ray source spot size and the sharper the X-ray image. The XMT system (MuCAT 2) developed at Queen Mary University of London (QMUL) is unique in that it employs time-delay integration (scanning a CCD camera through the X-ray beam with synchronous readout) to eliminate ring artefacts and to facilitate the creation of high contrast-ratio images using long exposures^6^. It should be noted that this system was used due to ease of access for the authors; the experimental work could have been performed on any number of commercial micro-CT or other microfocus X-ray systems. The QMUL scanner uses an X-tek (now Nikon Metrology UK Ltd), 225 kV X-ray generator and has a 100 µm thick CsI scintillator, fibre-optic coupled to a 6 cm × 6 cm CCD camera (Spectral Instruments, Inc., Tucson Arizona). The CCD has 15 µm square pixels, with 2 × 2 on-chip binning. Further 2 × 2 binning is performed after TDI readout to give a final pixel size of 60 µm. Geometric magnification gives a reconstructed voxel-size of 7.5 to 40 µm. The nature of the dynamic dental research performed with this system requires consistent performance and therefore constant attention to the X-ray focus.

The X-ray generator is of the demountable type and runs at a higher power than typical sealed-source systems. With continuous usage, filaments must be replaced approximately monthly, and each filament change involves a lengthy focus and alignment procedure. Furthermore, over the course of its lifetime, the filament becomes thinner. Without adjustment to the filament current, it will become hotter, and the lifetime will be reduced. Focus is very dependent on filament current and so re-focus is required every one to two days. Re-focus is also required if the accelerating voltage is changed. When only a low resolution is required, the filament current can be reduced to prolong the lifetime, but it will be necessary to check that the focus is sufficient. The magnetic lens^7^ in the X-ray generator requires not only a general focus setting, but also X- and Y-shift adjustment, the optimum values of which may drift with time. Non-optimal X- and Y-shift results in aberrations and thus the focussing procedure is far more complex than with a simple optical camera. The X-ray generator uses a cylindrical tungsten target with a threaded mechanism that allows it to be “indexed” to expose a fresh target region. Over time, the target becomes pitted which also affects the focus. Indexing of the target resolves this.

The manufacturer’s quoted focal spot size is down to 3 µm, although this would be at lower beam current than is used in our research where a high contrast-ratio is required. The method normally employed at QMUL to check the focus is to place a 3 mm diameter tungsten rod in the beam at a projected pixel size of 5.5 µm; the minimum size allowable leaving clearance between it and the filter/collimator assembly. An image is acquired, and the sharpness is quantified by measuring the mean (over the 1000 rows of the image) interpolated distance between the 40 and 60% intensity levels on either edge of the shadow. The focus, X-shift and Y-shift are manually, iteratively adjusted to minimise this distance (since they are interdependent, they cannot simply be individually optimised). If a sufficiently low value is not obtained, the filament current may be increased, and the process repeated. Similarly, if the value is lower than required for the intended imaging resolution, the filament current may be reduced, and again the procedure repeated. Although this technique only measures sharpness in the horizontal direction, it is affected by both X- and Y-shifts as either will cause aberrations if incorrectly set. However, it is not easy to tell which of these settings require adjustment and by how much. The TDI system takes approximately 10 s to acquire a single image; to obtain an optimal focus is a very slow process. The possibility of imaging the focal spot was therefore explored to give a better indication of the direction and magnitude of these aberrations and to quantify the spot size to allow for more consistent imaging. A simple method of focussing the X-ray system would also be of benefit to users of commercial micro-CT equipment who often rely on subjective assessment of focus.

The most direct method of imaging the focal spot is by means of a pinhole^8^. However, the pinhole needs to be smaller than the minimum spot size, which in this case is just a few microns. From simulated X-ray spectra^9^ it was determined that a lead thickness of 200 µm, or gold thickness of 120 µm would be needed to get even 80% attenuation of a 90 kV X-ray beam (the voltage typically used in our dental research). Not only would it be difficult to create and align a 3 µm pinhole in such a thickness of material, but this would have a collimating effect that would nullify its use as a focussing tool. Use of a slit^10^ would also be problematic. Imaging of the focal spot has also been performed using a coded aperture^11^, which uses a mathematically generated pseudo-random array of apertures, rather than a single aperture, to improve the signal-to-noise ratio (SNR). A requirement of the pseudo-random pattern is that a decoding array exists such that the correlation of the coded aperture pattern and the decoding array is a Dirac function (single point). An X-ray image of the coded aperture appears as multiple overlapping images of the X-ray spot. Correlation of this image with the decoding array (inverse of the coded aperture pattern) at the correct scale yields a single image of the X-ray spot with a much higher SNR than could be obtained with just one aperture. In the published example, the X-ray generator was from a mammography system and the coded aperture was 80 µm thickness of tungsten with 70 µm diameter holes. This thickness of tungsten was reported to give about 11% transmission at 35 keV, rising to around 31% at 80 keV. In the case of a microfocus X-ray tube, the SNR is limited not only by the lower beam current, but also by the fact that, due to the smaller hole size, the aperture material must be thin, both for practical reasons and to avoid any collimating effect, and thus the transmission will be much higher.

## Methods

### Scanner setup

**Figure 1:**
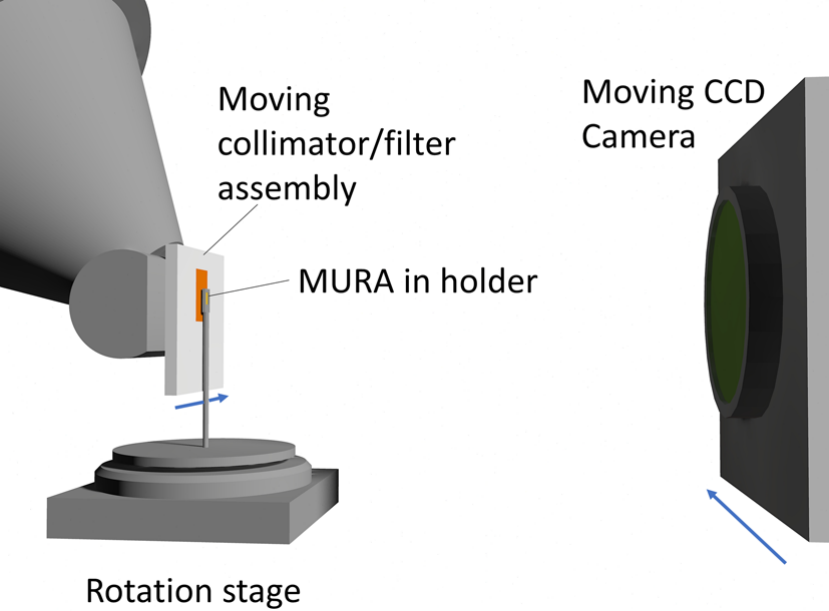
MuCAT 2 micro-CT scanner arrangement with the MURA mounted.

The arrangement of the MuCAT 2 scanner is presented in Fig. 1. The X-ray camera is positioned with the scintillator approximately 24 cm from the X-ray source, giving a beam divergence of approximately ± 7° in the horizontal and vertical direction. The camera moves across the X-ray field in order to generate a time-delay integration image (CCD rows are vertical and the charge image is shifted in the opposite direction to the camera motion such that it is static during readout). A filter/collimator assembly tracks the motion of the camera such that X-rays directed outside the imaging area are masked off (this reduces scattered radiation). In TDI mode, the maximum horizontal image size is 2500 pixels, limited by the X-ray output aperture. To obtain the right geometric magnification, the coded aperture is mounted 22 mm from the X-ray source (the position of the source is determined through a calibration procedure where a horizontally mounted tungsten rod is raised through the beam at known positions). For simplicity, the figure does not show the vertical motion stage that raises and lowers the sample, and the horizontal stage moving in the source-detector direction that changes the geometric magnification.

### Coded aperture specification

With a limit on the closest distance to the X-ray spot in the MuCAT 2 system, the minimum equivalent pixel size is 5.5 µm. Without the post-acquisition 2 × 2 binning, this becomes 2.75 µm although the resolution is limited by the scintillator thickness and the blurring caused by the continuous camera motion versus the stepwise charge motion in the CCD during TDI readout. Thus, a detail size of 2.75 µm was chosen which could be produced with a 5 µm gold thickness on a 200 µm thick silicon substrate. As well as avoiding collimation effects, the thin aperture thickness has the additional advantage that the relationship between transmitted intensity and path-length is very close to linear over such a short range (the beam is filtered by 1 mm of aluminium and 50 µm copper to attenuate soft radiation). Using the modelled spectrum, at 90 kV the expected attenuation of 5 µm of gold is approximately 12% (88% transmission). Thus, when the X-ray beam is not perpendicular to a hole, the edges will be blurred, but the overall transmitted intensity would not differ appreciably; this would not be the case with a thicker aperture. Since the area of the focal spot is much larger than the hole size, contrast values are likely to be of the order of 1% or less, meaning that images of individual holes would not be discernible from the noise.

### Pattern specification

The principle of coded aperture use is that multiple pinholes are used to increase the SNR, producing a multiplexed image^12,13^. From this, an image of the original object is then mathematically reconstructed. Fenimore and Cannon^14^ describe uniformly redundant arrays (URA) which have perfectly flat sidelobes, meaning that “whereas the inherent noise in random array imaging puts a limit on the obtainable SNR, the URA has no such limit.” Modified URAs (MURAs) were later introduced^15^ whereby the image is reconstructed by correlation with a unimodular decoding array. A further refinement of this is to intersperse the rows and columns of both the aperture and decoding arrays with zeros, creating a “no-two-holes-touching” array^16^. Although the aperture array is not self-supporting in this case, this approach was chosen to avoid non-linearities in transmission that could otherwise occur when adjacent holes merge. Note, in this text, the term “MURA” may refer to a mathematical concept or a physical device, depending on the context.

The height of the active image area is 2000 2 × 2 binned pixels. With interleaved zeros, the cell size for the MURA is 5.5 µm and at least one repeat is required for correlation. The sequence length for the array must be a prime of the form 4 m + 1^15^, giving a maximum of 461 (2.5355 mm). However, for the purpose of focussing, only an area of tens of microns across is of interest and so a much shorter sequence can be used. A shorter sequence can be repeated to fill the imaging area so that there is no loss of SNR. It can be decoded by correlation with the same repeated pattern, or by a single sequence in selected areas of the image and the resultant reconstructions averaged together. The pattern selected had a sequence length of 29, repeated 34 times in both dimensions to give a total area of 5.423 × 5.423 mm and having a total of 485,520 “holes”; the previous mammography system had only 480 holes^11^.

A single aperture array A for sequence length p is defined^15^ as:

1 $$A = \left\{ {A_{ij} } \right\}\begin{array}{*{20}c} {p - 1} \\ {i,j = 0} \\ \end{array}$$

With values set to:

2 $$A_{ij} = \left\{ {\begin{array}{*{20}c} 0 & {if \,i = 0,} \\ 1 & {if\, j = 0, i \ne 0,} \\ 1 & {if\, C_{i} C_{j} = + 1,} \\ 0 & {otherwise} \\ \end{array} } \right.$$

where:

3 $$C_{i} = \left\{ {\begin{array}{*{20}c} { + 1} & {{\text{if}}\;i\;{\text{is}}\;{\text{a}}\;{\text{quadratic}}\;{\text{residue}}\;{\text{modulo}}\,p} \\ { - 1} & {{{otherwise}}} \\ \end{array} } \right.$$

**Figure 2:**
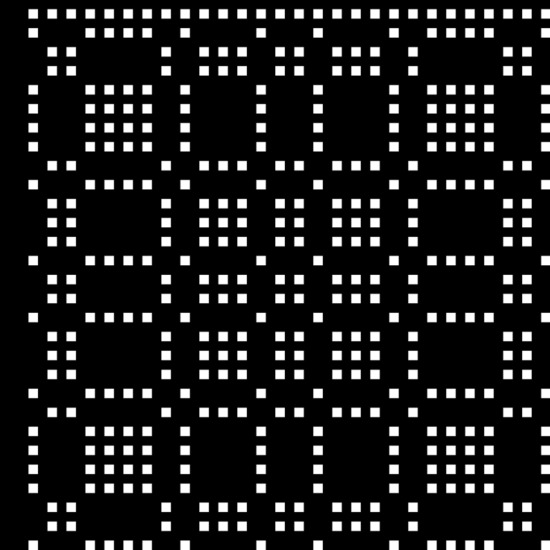
Single MURA array, with sequence length of 29. The white squares are fabricated as holes in the 5 µm thick gold.

This is shown in Fig. 2, where the white squares will become holes when fabricated.

The decoding array is given by:

4 $$G_{ij} = \left\{ {\begin{array}{*{20}c} { + 1} & {if\, i + j = 0,} \\ { + 1} & {if \,A_{ij} = 1, \left( {i + j \ne 0} \right),} \\ { - 1} & {if \,A_{ij} = 0, \left( {i + j \ne 0} \right)} \\ \end{array} } \right.$$

**Figure 3:**
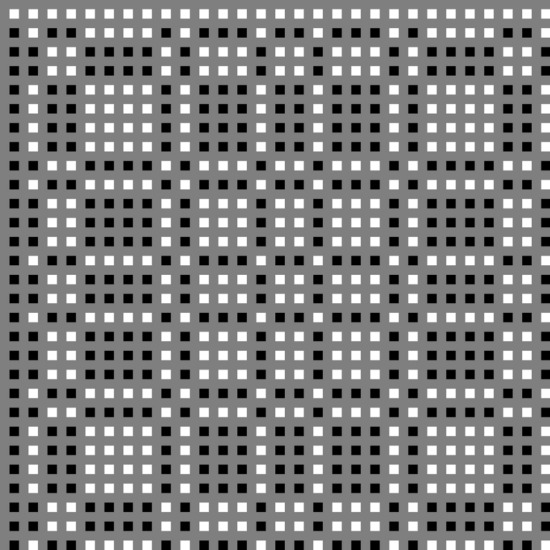
Visual representation of the decoding array used to reconstruct the X-ray spot image. Black is − 1, grey is zero, and white is + 1.

This is visualised in Fig. 3, where black represents -1, grey is 0 (interspersed values) and white is + 1.

### Fabrication

**Figure4:**
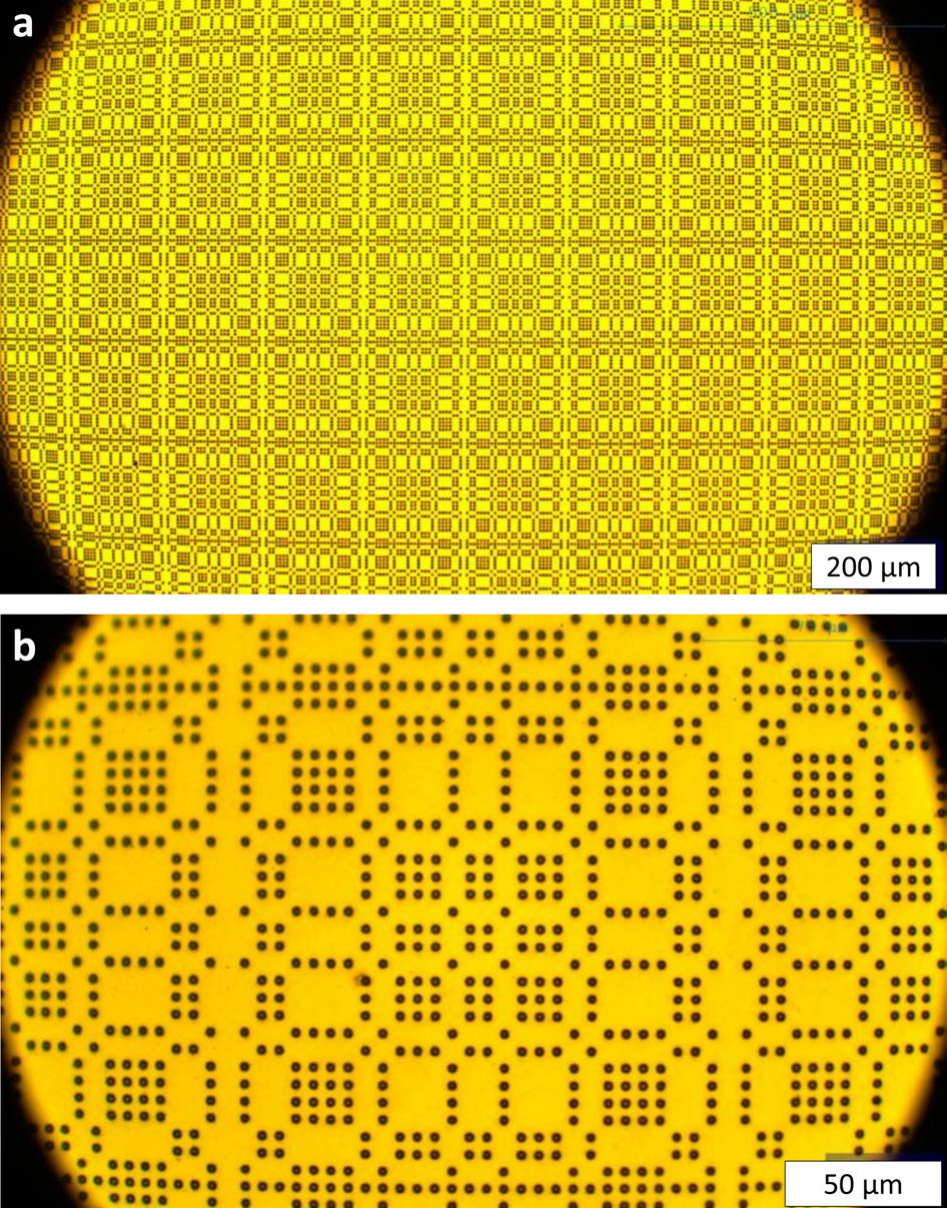
Exemplary optical microscope pictures of a MURA. (**a**) overview, (**b**) detailed.

**Figure 5:**
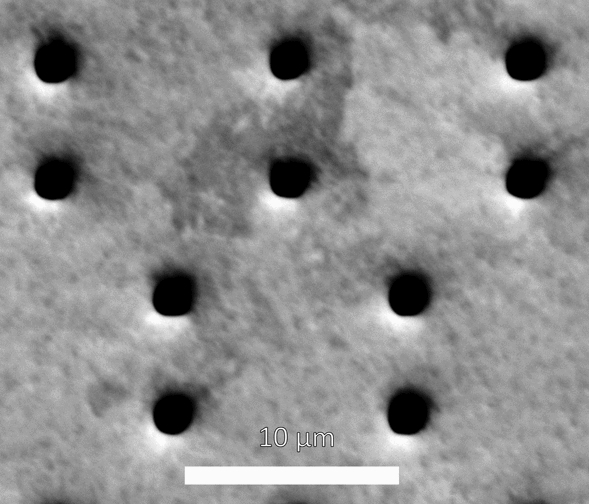
Back-scattered scanning electron microscope image of the MURA detail taken with a Zeiss EVO MA10 scanning electron microscope.

As mentioned previously, the aperture material must be thin; 5 µm gold thickness was chosen. Fabrication was performed by the Microworks GmbH company (Karlsruhe, Germany) and the Institute of Microstructure Technology (Karlsruhe Institute of Technology, Karlsruhe, Germany). The Direct Laser Writing process^17^ (DWL66 + from Heidelberg Instruments, Heidelberg, Germany) was performed to pattern the 2D structure; it allows the processing of the needed minimum feature size (2.75 µm) using a substrate size up to 6″. A 200 µm 4 inch silicon wafer with a CrAu thin layer was first coated with a 100 nm bottom antireflection coating (AZ Barli, MicroChemicals GmbH). A 7 µm epoxy-based photo resist layer was then spin coated and written with a focused laser beam (wavelength 355 nm). No mask is needed as in conventional photo lithography. After a post exposure bake (PEB) step, the resist was developed in a 1-MethoxY-2-propyl-acetat (PGMEA) solution for 20 min and rinsed using isopropanol. A reactive ion etching (RIE plasma etcher Etchlab 200, SENTECH Instruments GmbH) sequence was performed to remove the antireflection layer, before conducting the gold electroplating step. A height of 5 µm gold (+ /− 0.5 µm) was deposited in the opened structure. The resist was removed using a plasma stripping tool (R3T/MUEGGE GmbH). The wafer was then diced to separate the different MURA pieces. In Fig. 4, exemplary optical microscope pictures of a final product are presented. A back-scattered electron image, taken with a Zeiss EVO MA10 scanning electron microscope, is shown in Fig. 5. It can be observed that the apertures are round, rather than square, and only approximately 2 µm in diameter. This known effect of corner rounding of the square designed structure is due to the diffraction limitations and other processing effects such as PEB (Post Exposure Bake) diffusion.

### Image acquisition

**Figure 6:**
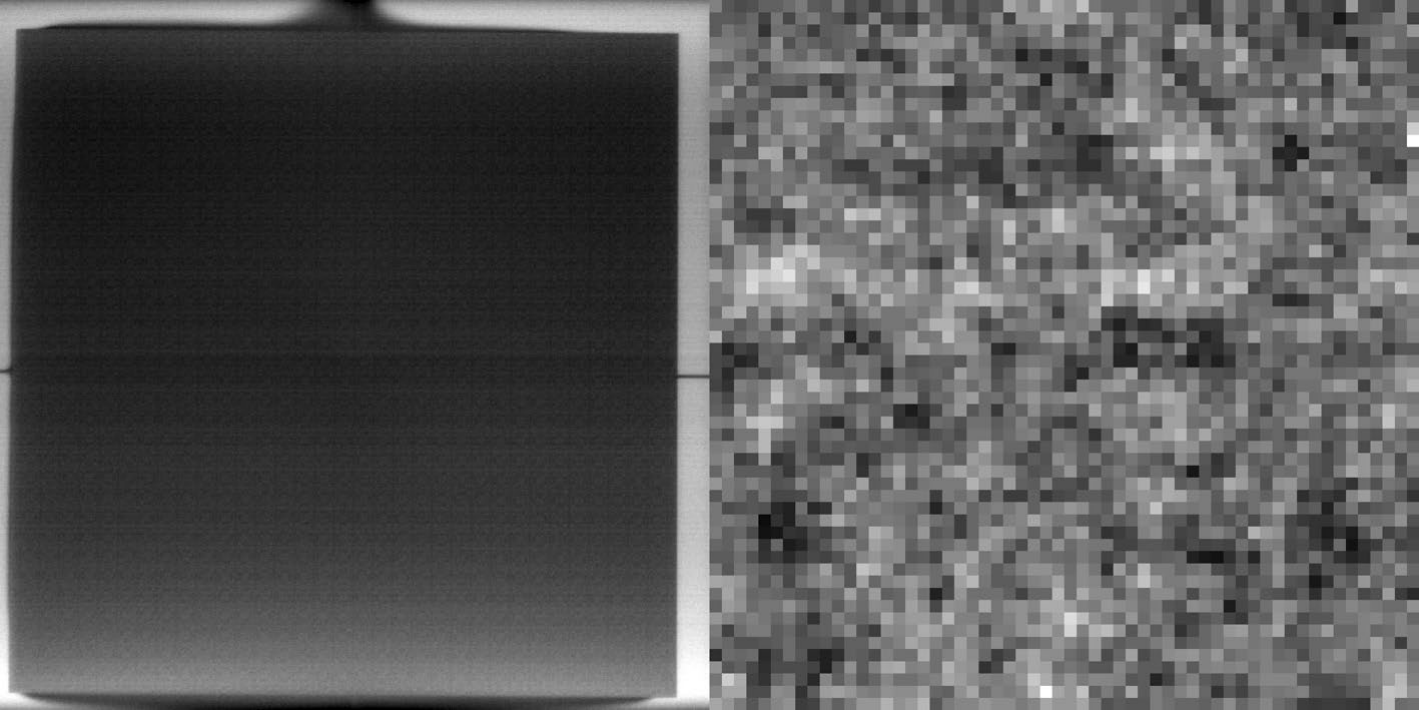
Left: Single image of the repeated MURA pattern with an approximate effective 2.75 µm pixel size. Right: Magnified view showing a 58 × 58 pixel region representing a single MURA sequence.

The X-ray generator voltage was set to 90 kV and current to 180 µA; typical settings for the dental research for which it was designed. The exposure time was set to 3.3 s, the shortest available, representing the time taken for the CCD to traverse its own width; the actual acquisition time is approximately 10 s including camera flyback. The recorded TDI image was 2040 X 2000 pixels, without the usual post-readout 2X2 binning, i.e., having a 30 µm pixel size. The resolution is thus limited by the scintillator, so any image of the source will be correlated with this detector blurring. The MURA was positioned to allow geometric magnification that gave an effective pixel size of approximately 2.75 µm at the sample position (note, for source to sample distance *S* and source to detector distance *D*, magnification of the sample is *D*/*S* whilst magnification of the X-ray spot is (*D-S*)/*S*). Initial dark and light field images (X-rays off and on with nothing in the beam) were taken so that subsequent images could be normalised (flat-field correction; these do not need to be taken repeatedly). A sample image (without flat-field correction) is shown in Fig. 6 (contrast has been expanded to fill the dynamic range). Note, because of TDI imaging, the effects of scintillator and detector inhomogeneity are smoothed in the horizontal direction only. The mean attenuation through the MURA was approximately 10% compared with the substrate. A 58 × 58 pixel region is shown on the right, representing a single MURA sequence. Because of the low SNR, no clear resemblance to the original pattern is apparent.

**Figure 7:**
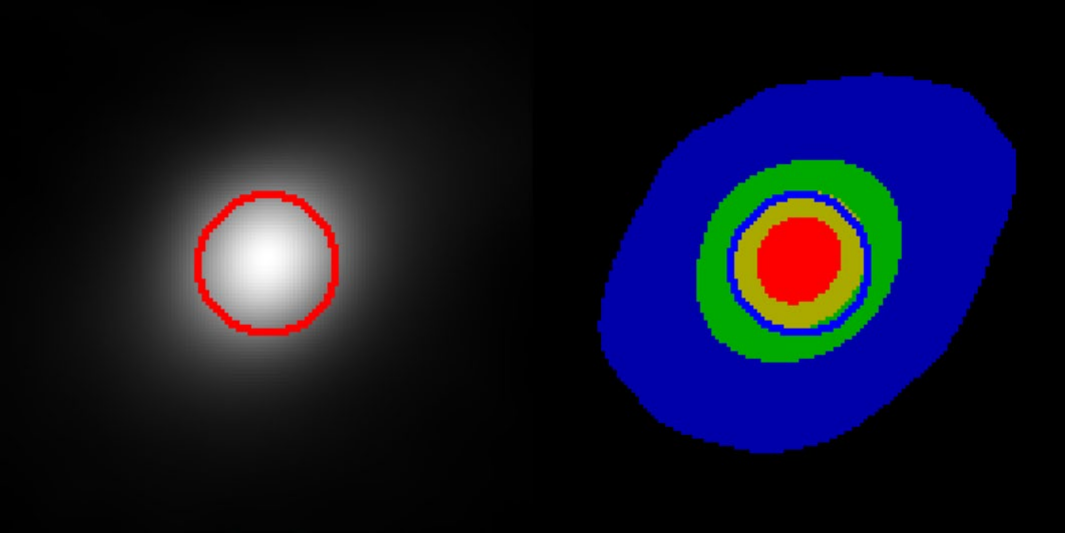
Output from the prototype software showing an image of the focal spot. Left: Monochrome image with a 10 µm red circle for scale. Right: Colour image where red is 75–100% peak, yellow 50–75%, green 25–50% and blue 5–25%, with blue 10 µm diameter blue circle.

Prototype analysis software was written in Interactive Data Language (IDL, from Harris Geospatial, Colorado, USA). The MURA was held in a simple 3D-printed holder and perfect orientation could not be guaranteed. The rotation angle of the micro-CT turntable was adjusted so that the image of the MURA was square, rather than trapezoidal. When the analysis software first runs, the user is prompted to click on the left and right edges of the MURA so that an initial scale can be set. A 232 × 232 central region is then correlated with a 4 × 4 repeat of the decoding array (this was found to be more sensitive than using a single sequence). Because of the repetitions, the correlation output is also repeated and can be cropped to 58 × 58 pixels. Scale and rotation of the MURA image are then adjusted using a simplex optimisation algorithm^18^ that maximises the peak of the 2D correlation output. The rotated and scaled image is then divided into 8 × 8 tiles, each 232 × 232 pixels. To allow for slight errors in the rotation and scale, each tile is correlated with the decoding pattern and the location of the centroid for each is measured. These location coordinates are smoothed by fitting a 2^nd^ order 2D polynomial such that the centroids for all 64 correlation outputs can be aligned and summed (the output is shifted and wrapped such that the centroid of the averaged focal spot image is at the centre). In this prototype software, the image is then upscaled by a factor of 10 with linear interpolation, cropped to 500 × 500 pixels and displayed to the user. The greyscale is set so that zero intensity is black and the peak intensity is white. A 10 µm diameter circle is overlaid in the centre. An example output is shown in Fig. 7. To the right of the greyscale image is a colour image, where 75–100% peak intensity is shown in red, 50–75% in yellow, 25–50% in green, and 5–25% in blue (a 10 µm diameter circle is also displayed in blue). This latter blue band is useful for showing low intensity aberrations. In order to obtain a quantitative measure of focus that gave a similar metric to that obtained with the tungsten rod, the columns of the output were summed, and a cumulative distribution calculated. The distance in microns from 25 to 75% intensity was then calculated. For the output illustrated in Fig. 7, this value was 7.35 µm. After obtaining a best focus, offsets were made to the X-shift, Y-shift, focus, and filament current. Further tests were carried out at 120 kV and 135 µA with 0.44 mm Cu filtering, then at 160 kV and 100 µA with the same filtering. The X-ray generator uses a cylindrical tungsten target with a threaded mechanism that allows it to be “indexed” to expose a fresh target region.

The SNR was calculated as the ratio between the peak intensity and the standard deviation of the background away from the spot. This value was not routinely presented to users for focussing purposes but was calculated to test the validity of the technique. For comparison, an estimate of the SNR from a single aperture was made from the known attenuation of the gold, the measured spread of the intensity, and noise from a “light” image (computed from the difference between two successive images).

The method was also tested without dark and flat-field correction. The result exhibited some background patterning due to variations in intensity. To eliminate this, columns were averaged together outside the focal spot region and the averaged column value was subtracted for each corresponding row, and vice versa.

## Results

The IDL software took around 3 to 4 s to calculate the scale and rotation angle of the first image and then around 1 s to compute the X-ray-spot output from subsequent images. Note, IDL is a fourth generation language and was chosen because of the ease with which image analysis algorithms can be prototyped; execution speed is slow in comparison with other development platforms. This time is still short in comparison with the image acquisition time of 10 s. The required rotation angle was less than 0.1° and magnification 1.003; very close to unity.

**Figure 8:**
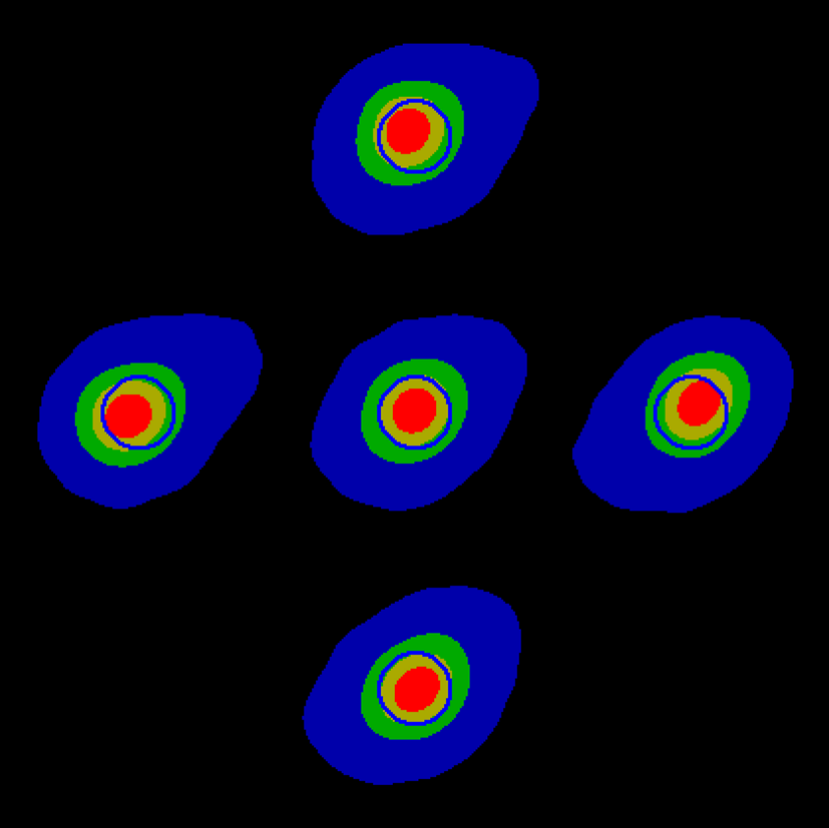
Effect of X- and Y-shift (see Fig. 7 for colour scale). Centre: Optimal. Left: X-shift -20. Right: X-shift + 20. Top: Y-shift − 20. Bottom: Y-shift + 20.

**Figure 9:**
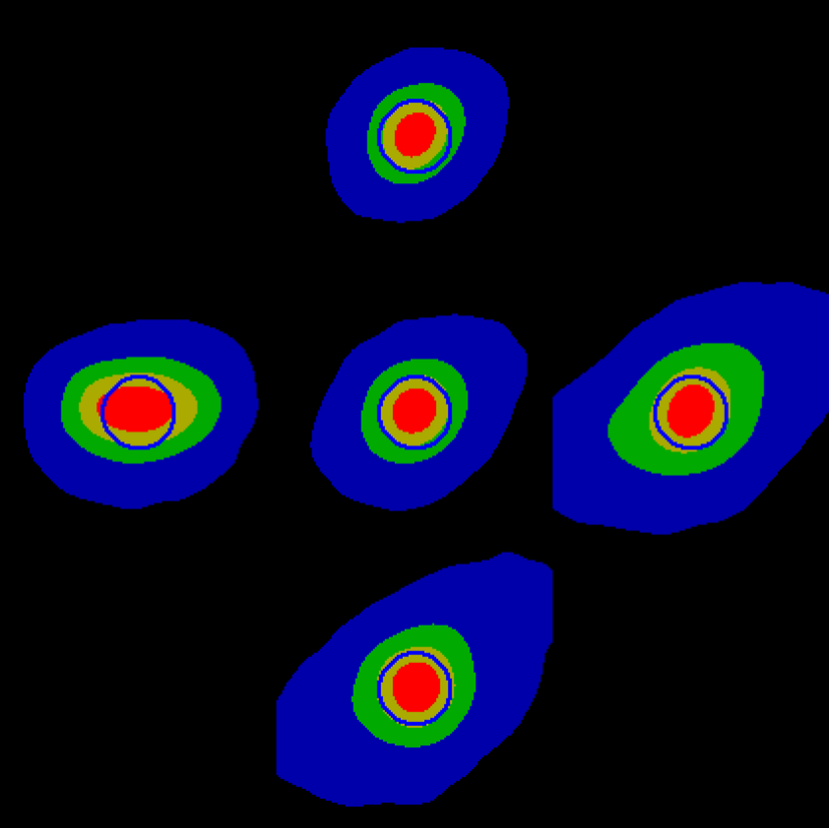
Effect of adjusting focus value and filament current (see Fig. 7 for colour scale. Centre: Optimal. Left: Focus -10. Right: Focus + 10. Top: Filament current + 5. Bottom: Filament current − 5.

Figure 8 shows the effect of adjusting the X- and Y-shifts. Because the images are centred on the centroid, and this is affected by the aberrations, the peak appears to move as the X- and Y-shifts are altered. Shift values are in the range of + / − 125 arbitrary units which control electromagnetic deflection. Further shifting is available using mechanical screws to move the electron gun. Increasing X moves the peak to the right and increasing Y moves it downwards, though there appears to be an approximate 30° rotation. The best focus value is obtained when the peak is centred using the X- and Y-shift (see Table 1). Having a visual indication of the direction in which X- and Y-shifts need to be adjusted makes focussing far more intuitive and faster. A better focus is seen when the filament current is increased by 5 units, though this would reduce the filament lifetime (Fig. 9). The optimal value is considered sufficient for micro-CT scans performed with a 15 µm voxel size; the value normally used in the authors’ dental research. Reducing filament current makes the focus worse but will prolong filament life when high resolution is not required. The effect of changing the focus setting is also clearly seen. At higher voltages (120 and 160 kV), the focal spot was clearly visualised, with some discernible noise in the 160 kV image. Using the background correction method (above), images obtained without dark and flat-field correction were of the same quality.

**Table 1:** Focal spot width measured in the X-direction as a function of X-shift, focus value (set on the X-ray control system), and filament current.

Setting (arbitrary units)	Focus (µm)
Optimal	7.35
X-shift − 20	7.61
X-shift + 20	7.37
Y-shift − 20	7.62
Y-shift + 20	7.50
Focus − 10	9.33
Focus + 10	9.33
Filament current − 5	8.29
Filament current + 5	6.69

For the standard setting of 90 kV and 180 µA, the measured SNR was 376. This fell to 138 for 120 kV and 135 µA and to 130 for 160 kV and 100 µA. Further tests were carried out with reduced current. At 90 kV and 5 µA, the measured SNR was 96.

## Discussion

The most important output from the technique is visual feedback of the X-ray spot showing spread and aberrations. With four degrees of freedom (X-shift, Y-shift, focus and filament current), manually focussing the X-ray system is a skilled and time-consuming process. The offsetting of the peak with respect to the centroid gives a clear indication of the direction in which shifts must be made to improve the focus. A 30° rotation of the output image would make an even more direct correspondence between shift adjustment and peak position; the reason for this 30° rotation is not clear and requires further investigation. A sequence length of 29 × 29 was chosen because this should adequately cover the spread of the X-ray spot. However, the SNR from decoded images from a single pattern did not allow precise identification of the peak intensity position, and thus 4 × 4 repeated MURA sequences were used. The larger the size of the pattern, the more precise must be the determination of magnification and rotation, as well as any corrections for non-perpendicular orientation of the MURA. The MURA sequence length could be increased to 113 and then a single pattern would give a similar SNR to the 4 × 4 block and would increase the coverage for the focal spot imaging. When the background correction method was used, the coverage is reduced further as the column and row averaging must be performed in areas outside the focal spot image. However, the sequence length of 29 (129.5 µm) proved to be more than sufficient in this case.

At higher voltages there was little loss of quality in the images, however the detected X-ray spectrum is limited by the thickness of the scintillator, which was not optimal for these higher voltages, giving much greater sensitivity to lower energy photons. For this reason, the results are not reported quantitatively here. Target pitting could be seen as abnormal aberrations or an abnormal relationship between X- and Y-shift and movement of the peak. This was resolved by indexing the target. In this way, pitting was found to be occurring more rapidly than we had previously thought and is not surprising for the power setting used.

At standard X-ray settings, the SNR of 376 was more than adequate, giving a high-quality image of the X-ray spot. The estimated SNR for a single aperture was 1.5, meaning that it would not be visibly discernible from the noise. Since an array of 8 × 8 MURA sequences was used, a more reasonable comparison would be with an 8 × 8 set of apertures with the images averaged together. This would give an SNR of 12, meaning that the spot would be visible, but it would be difficult to discern, let alone quantify the aberrations. At 5 µA beam current, the SNR of 96 given by the MURA would still be adequate for this purpose and provided a clear image. At this current, the SNR from an 8 × 8 array of apertures was estimated at 3.2. At both current settings, use of the MURA gave an SNR approximately 30 times higher than that estimated for an 8 × 8 array of single apertures.

The round, rather than square aperture shape (Fig. 5) had no discernible effect on functionality, however, the cross-sectional area of the holes is a factor of 2.4 times lower than that of the ideal shape, thus reducing the SNR. To obtain a better accuracy design-copy, the use of another process like electron beam lithography, combined with X-ray lithography is possible; a more economical approach would be to simply increase the size of the designed aperture to yield a physical aperture of the required cross-sectional area.

The detector resolution in the QMUL microtomography system is limited by the thickness of the scintillator. There will be some horizontal blurring due to the time-delay integration readout and further blurring due to interpolation when the image is magnified and rotated. This latter source of blurring could be eliminated by physically rotating and moving the MURA rather than transforming the image, but this level of blurring is very low compared with that caused by the scintillator thickness and is small in comparison with the X-ray spot size.

## Conclusion

The methodology presented here was used in conjunction with a unique TDI X-ray imaging system, but is applicable to any microfocus system of the types commonly used in micro-CT. Although the software and the physical MURA holder were at a very early stage of development, we demonstrate that the use of a coded aperture based on repetitions of a modified uniformly redundant array has greatly simplified the process of adjusting and quantifying the focus of a micro-focus X-ray source used in micro-CT. A rigid, precision holder would eliminate the need for rotation and magnification checking and GPU coding would allow much faster processing. Reducing the beam current by a factor of 36 still yielded a satisfactory SNR. This implies that at the normal beam current, the exposure time could be reduced from 3 s to less than one tenth of a second, allowing real-time imaging of the focal spot with a suitable detector. Currently, visualisation of the X-ray spot allows an estimate to be made of the required X- and Y-shift adjustment. In the future, quantification of the direction and magnitude of aberrations could provide feedback for automatic focussing.

The fabrication challenge for focus aids for microfocus X-ray sources is that as the spot gets smaller, smaller patterns are needed with a sufficient absorption contrast. Using a “simple” and “cheap” fabrication process (Direct Laser Writing), this study, involving fabrication of 2.75 µm × 2.75 µm structure with 5 µm thick gold, has proved that a good image of the X-ray spot can be obtained even with a thin material that gives a very low contrast image. Moving to a more complex and expensive process like electron beam lithography, combined with X-ray lithography, a much smaller feature size could be realized, allowing smaller focal spots to be imaged as are attainable with transmission target X-ray systems. At the current scale, no diffractions effects were apparent, though this may become an issue at smaller scales.

## Data Availability

MURA raw images were taken with a non-standard format and were not stored. However, Tiff images can be made available on reasonable request to Graham Davis at Queen Mary University of London.

## References

[1] Schladitz K (2011). Quantitative micro-CT. J. Microsc..

[2] Keklikoglou K (2021). Micro-CT for biological and biomedical studies: A comparison of imaging techniques. J. Imaging.

[3] Morigi MP (2010). Application of X-ray computed tomography to cultural heritage diagnostics. Appl. Phys. A.

[4] Cunningham JA (2014). A virtual world of paleontology. Trends Ecol. Evol..

[5] Swain MV, Xue J (2009). State of the art of micro-CT applications in dental research. Int. J. Oral Sci..

[6] Davis GR, Evershed ANZ, Mills D (2013). Quantitative high contrast X-ray microtomography for dental research. J. Dent..

[7] Hawkes P, Kasper E (2017). Magnetic lenses. Principles of Electron Optics.

[8] Arnold BA, Bjarngard BE, Klopping JC (1973). A modified pinhole camera method for investigation of X-ray tube focal spots. Phys. Med. Biol..

[9] Poludniowski G (2009). SpekCalc: A program to calculate photon spectra from tungsten anode x-ray tubes. Phys. Med. Biol..

[10] Rong XJ (2003). Measurement of focal spot size with slit camera using computed radiography and flat-panel based digital detectors. Med. Phys..

[11] Russo P, Mettivier G (2011). Method for measuring the focal spot size of an x-ray tube using a coded aperture mask and a digital detector. Med. Phys..

[12] Dicke RH (1968). Scatter-hole cameras for X-rays and gamma rays. Astrophys. J..

[13] Blake RL (1974). Solar x-ray photography with multiplex pin-hole camera. Rev. Sci. Instrum..

[14] Fenimore EE, Cannon TM (1978). Coded aperture imaging with uniformly redundant arrays. Appl. Opt..

[15] Gottesman SR, Fenimore EE (1989). New family of binary arrays for coded aperture imaging. Appl. Opt..

[16] Fenimore EE, Cannon TM (1981). Uniformly redundant arrays: Digital reconstruction methods. Appl. Opt..

[17] Achenbach S (2018). Polymer-based X-ray masks patterned by direct laser writing. Rev. Sci. Instrum..

[18] Nelder JA, Mead R (1965). A simplex method for function minimization. Comput. J..

